# The Problems of Private Practice
*Mr. "Stephen Andrew's" first article was published in The Hospital of June 3.


**Published:** 1911-06-17

**Authors:** Stephen Andrew


### MEDICAL PROBLEMS THAT PRESS.

### THE PROBLEMS OF PRIVATE PRACTICE.

### II.—HOSPITALS: FROM THE POINT OF VIEW OF THE G. P.*

By STEPHEN ANDREW.

Among the devices for the relief of those who are sick, and at the same time poor, the voluntary hospital occupies an honourable and certainly a very ancient place. Unfortunately hospitals, like other ancient institutions, are finding it more and more difficult to keep up appearances under modern conditions; and the inevitable consequence is that they are steadily becoming unable to do the work they profess to do. As often as not they cannot grow in proportion to the growth of the population round about them, while it not infrequently happens that they are, in part, at any rate, understaffed and under-equipped.

As a rule, the in-patient department of a British hospital is excellent in every respect, except size. The only criticism to be offered, in the huge majority of cases, is "Not enough beds." Wards, operating theatres, equipment are as a rule very good indeed. The patients get adequate medical and surgical treatment, competent nursing, good food; and if there were beds enough to accommodate all who are in urgent need of in-patient hospital treatment there would be little to complain of.

#### The Tradegy of Out-Patients.

The out-patient department of many hospitals, on the other hand, is often extremely unsatisfactory. There, patients are put to quite unnecessary inconvenience, discomfort, and even danger; while the medical and nursing staffs are called upon to work under conditions which are difficult and often very disquieting.

It is amazing that patients put up with the treatment they receive in the out-patient department of many hospitals. To begin with, they have to arrive at the hospital at some fixed hour, which is often uncomfortably early. This may be a small matter to those whose homes are close to the hospital, but it is anything but a small matter to those who come from a distance. It means, not infrequently, that the unfortunate mother of a sick child is forced to hurry off with her invalid to catch an early train, leaving the rest of her children to get off to school on their own account—often without any breakfast. Then, arrival by the fixed time does not by any means result in prompt attention and speedy release. On the contrary, the average out-patient may count upon a wait of several hours in an uncomfortable, evil-smelling, draughty waiting-hall. He will be one of a great mass of closely-packed sick persons, many of whom will be dirty—some offensively so; many of whom will be suffering from diseases which are openly repulsive—such as lupus, impetigo, or some other skin affection; some of whom may be vigorously infectious. The out-patient must take his chance. Luck may give him for neighbours several clean, pleasant folk, or it may provide him with a man with scabies on his right, a woman with pediculi on his left, a child with scarlet fever in front, and a youth in an advanced stage of consumption behind. Luck again may carry him into the room of the doctor soon, or it may keep him out of it until the end of the morning. In the first case—if his case be interesting—he will receive very careful and thorough attention; in the second, he will not receive quite so much care, for the doctor will be fagged and anxious to be done with his work.

#### The Woes of Out-Patients.

Then, after the ordeal of the waiting-hall and the doctor's room, the patient will not be through with his troubles. He still will have to wait for his turn at the dispensary, whence he will bear away an enormous bottle of medicine, and it may be that his turn will be a very long time in coming

From first to last it is no unusual thing for a visit to the out-patient department of a big hospital to consume the greater part of a working day, and that, to a working-man, or a working-man's wife, is an extremely serious matter. In the case of the man it means that the advice and medicine he has received—gratis and from a voluntary charity, be it remembered—has cost him a sixth part of his week's earnings. This is rather a big price to pay, especially when all the inconveniences and other drawbacks are taken into consideration.

That the medical and nursing staffs in the out-patient department are often called upon to work under hopelessly unsatisfactory conditions is well known to numbers of hospital doctors and nurses. Unfortunately it seldom occurs to them to say so. Case after case comes into the physician's or the surgeon's room, obviously unfitted for out-patient treatment. There are three alternatives: (1) To admit the patient to a ward; (2) to send the case away for treatment by some general practitioner; (3) to tinker with it as an out-patient, with the certain knowledge that the tinkering is exceedingly likely to do more harm than good. If there is a vacant bed, well and good; but when, as often happens, there is no bed, the doctor is left to choose one of the other two courses. It is a pity that so often he decides on the third; for in so doing he is acting unfairly to the patient, to some general practitioner, and to himself.

He is unfair to the patient, for he is letting the latter think that he is being efficiently treated—which is far from being the case. Advantage is taken of the patient's ignorance—for the out-patient, like all other patients, is distressingly ignorant as regards his sickness and the best way of dealing with it—to palm off on him something which quite likely is not worth having.

He is unfair to the general practitioner, who might, if the patient were told that the out-patient depart-

* Mr. "Stephen Andrew's" first article was published in The Hospital of June 3.

ment was no place for him, earn a little money in return for efficient treatment of the patient.

He is unfair to himself, in that he is acquiescing in a line of action which he cannot defend on any ethical ground, and gradually drifting towards the point of view which regards an out-patient merely as an out-patient—a poor person, of no particular importance.

However, the ethics of the out-patient department and the relation of the out-patient staff to the patients call for a special article. The subject is too big to be treated in any way fully just at present.

One big drawback to the out-patient system is that the members of the medical staff, in London and the big provincial towns, are consultants, who are out of touch with the home life of poor people, and consequently know little or nothing of the conditions under which these out-patients are living. A result of this is that much of the advice given in the out-patient consulting rooms is absolutely wasted, for the patients cannot possibly act upon it. In circles where general practitioners move—particularly in places where they smoke pipes and swap yarns—there is often much ribald laughter at the expense of some hospital person or other. So often, the hospital man has forgotten that the out-patient is living in a condition which is very much like destitution, and has failed even to attempt to make his advice fit in with the patient's ability to take it.

General practitioners have to do much of their work under very difficult conditions. They, like the hospital men, are handicapped by the poverty of many of their patients; but they, at any rate, are fully alive to the conditions. Consequently they can, in a rough and ready way, do a great deal more for their patients than the out-patient medical-officer who has never seen the inside of his patient's home.

There is an enormous amount of work which can be done better in the out-patient department of a hospital than anywhere else. Every general practitioner cheerfully admits this, and sends suitable patients along to the hospital when he comes in contact with them. On the other hand, the out-patient rooms of hospitals are overcrowded because numbers of unsuitable cases come again and again, and never get told that they would be better if they never came near the hospital.

"Let 'em all come," as a hospital motto, may seem very nice and pretty and charitable; but consider the heavy hip disease child who is lugged for miles in the arms of some feeble woman; the consumptive girl who comes regularly for her bottle of Mist. Gent. c. Soda and flagon of Ol. Morrhuæ, and goes away to share a bedroom with three smaller sisters; the old woman with varicose eczema who needs rest in bed and cannot get it; the workman with a dilating right heart, who gets his bottle of medicine and goes off to strain his heart further at some heavy job; the dyspeptic whose dyspepsia is based upon chronic starvation, and is tinkered with a bottle of bismuth and sod. bic. . . .

This article ought to have been headed, "The Tragedy of the O.P.D."

